# Differences in Family Planning and Fertility Among Female and Male Gynecologic Oncologists

**DOI:** 10.1089/whr.2020.0046

**Published:** 2021-04-08

**Authors:** Mihae Song, Katelyn Tessier, Jani Jensen, Phoebe Leonard, Melissa A. Geller, Deanna Teoh

**Affiliations:** ^1^ Division of Gynecologic Oncology, Department of Obstetrics, Gynecology, and Women's Health, University of Minnesota, Minneapolis, Minnesota, USA.; ^2^ Biostatistics Core, Masonic Cancer Center, University of Minnesota, Minneapolis, Minnesota, USA.; ^3^ Reproductive Medicine and Infertility Associates, Woodbury, Minnesota, USA.

**Keywords:** family planning, fertility preservation, gynecologic oncologists, infertility, work-life balance

## Abstract

***Background:*** The objective of the study was to compare family planning and infertility among female and male gynecologic oncologists in the United States

***Methods:*** This cross-sectional multiple choice survey was administered to the Society of Gynecologic Oncology gynecologic oncologists. The survey collected information on demographics and practice, family planning, and fertility and infertility experiences. Chi-square and Fisher's exact tests were used to compare experiences by gender.

***Results:*** Two hundred eighteen of 1243 (18%) members responded to the survey. The majority were women (71%), Caucasian (78%), and had been practicing fewer than 10 years (56%). One-third (32%) were 35+ years of age at the birth of their first child, and 67% delayed childbearing due to their career. Women were more likely than men to report career choice-influenced family planning. Just under half (44%) expressed current or past concerns about fertility, and this was more prevalent among women; 81% had sought infertility counseling. Among respondents who had fertility struggles, almost half (45%) reported their colleagues were unaware. Forty percent felt their fertility concerns affected work life, and 13% felt stigmatized for their fertility struggles.

***Conclusions:*** These findings suggest that a career in gynecologic oncology have an impact on family planning, often resulting in childbearing delays and infertility concerns, especially among women. Support for our colleagues struggling with infertility should be included in wellness initiatives.

## Introduction

In 2017, for the first time in history, more women (51%) entered United States medical schools than men.^1^ This gender shift of the medical profession raises important questions about family planning, as most female physicians will spend a majority of their optimal reproductive years in medical training. Medical training, and especially surgical training, is stressful, and pregnancy and infertility add further physical and psychological burdens to trainees.^2,3^ Studies have shown higher incidence and severity of pregnancy complications among medical and surgical residents, including spontaneous abortions and preterm birth.^4,5^ More than 50% of female surgeons delay childbearing until they are in independent practice.^2^ In a 2000 survey of female gynecologic oncologists, a majority (62%) had their children after completing fellowship, and 75% of the respondents thought time after fellowship was regarded as an ideal time to have children.^6^

While fertility experiences have been studied among female medicine physicians and surgeons, there are limited data specific to gynecologic oncologists regarding infertility experiences. Unlike other surgical specialties, a majority of practicing obstetricians and gynecologists are female (57%), compared with 21% of general surgeons, and even smaller proportions of other surgical subspecialists.^7^ Moreover, this number is increasing, with females comprising 76% of gynecologic oncology fellows in 2018. Gynecologic oncology is unique in that an intensive surgical and medical oncology practice follows an obstetrics and gynecology residency, during which infertility risks and pregnancy complications associated with advancing age and infertility treatment options are core knowledge.

In the setting of a growing number of female gynecologic oncologists and the reproductive challenges they face, the primary objective of this study was to describe the differences in fertility and infertility experiences of female and male gynecologic oncologists. Secondary objectives were to assess the impact a gynecologic oncology career has on family planning decisions, and how fertility struggles in turn affect work life.

## Methods

### Study design

This cross-sectional survey study was approved by the Institutional Review Board at the University of Minnesota (STUDY00000781). Documentation of written informed consent was waived by the Institutional Review Board for this minimal risk and anonymous study. The study consent form was presented to all potential study participants, and clicking on the link to initiate the survey served as consent to participate in the study. A 35-item multiple choice online survey was beta tested with a voluntary cohort and subsequently revised; formal validation studies were not performed for this descriptive survey. The survey was sent by email in August 2017 to Society of Gynecologic Oncology gynecologic oncologist physician members, including fellows-in-training, who at the time of the survey practiced primarily in the United States; advanced practice providers, nongynecologic oncologist society members (*e.g.*, radiation oncologists, medical oncologists), and those practicing outside of the United States were identified by the Society of Gynecology based on self-provided membership information and excluded from the survey invitation email list. An initial survey invitation was sent followed by two reminder emails each 1 week apart. Study data were collected anonymously and managed using Research Electronic Data Capture (REDCap), a secure Health Insurance Portability and Accountability Act-compliant database which is compatible with mobile devices.^8^

### Survey

The survey collected the following: (1) demographic data: age, gender, race, and sexual orientation; (2) practice information: number of years in practice, geographic location at the time of first child, and practice setting at the time of first child; (3) family planning information: number of children planned compared with actual number of current children, desire to have additional children and potential ability to have more children at the current stage of life, whether childbearing was delayed due to profession, reasons for delaying childbearing (professional, financial reasons, personal, other), and whether career choice influenced number of children; (4) reproductive experience: number of pregnancies and outcomes, longest interval between trying to conceive and conception or decision to stop trying, and age at first delivery; (5) infertility experience: whether or not infertility assistance was sought, stage in training/career at which fertility assistance was sought, types of infertility treatment(s), and reasons for not seeking infertility treatment; (6) fertility preservation: consideration of or consultation for oocyte or embryo cryopreservation; and (7) emotional and psychological effects of infertility and infertility treatment: stigmatization, depression, effect on work life, and support from colleagues/administration (Supplementary Appendix S1). We asked respondents to answer questions as they pertained to their specific relationship(s), acknowledging that reproductive lifespan may be most dependent on the fertility potential of their partner(s) rather than the individual respondent. A free text area was provided for respondents to write additional comments.

### Statistical analyses

Demographics and other survey information were summarized for all survey participants using median and range for continuous variables, and frequencies and percentages for categorical variables. Information on reproductive planning and experience, fertility experience and treatment, and infertility support and psychological impact were also summarized for all survey participants and by gender using these measures. To investigate the association between gender and variables related to reproductive planning and experience, fertility experience and treatment, and infertility support and psychological impact, Wilcoxon rank-sum tests were used for continuous variables, and Chi-square or Fisher's exact tests were used for categorical variables, when appropriate. All reported *p*-values are two-sided and a significance level of 0.05 was used. Statistical analyses were performed in SAS (version 9.4; SAS Institute, Inc., Cary, NC).

## Results

### Demographics

Surveys were sent to 1243 gynecologic oncologists, and 218 surveys were completed, resulting in an 18% completion rate. A majority of respondents were women (71%), between 26 and 45 years of age (73%), and Caucasian (78%). Ninety-two percent of respondents identified as heterosexual, and 93% reported being currently partnered (Table 1).

**Table 1.** Participant Demographics (*N* = 218)

Variable	*n* (%)
Current age	
26–35 years	59 (27.1)
36–45 years	101 (46.3)
46–55 years	34 (15.6)
>55 year	24 (11.0)
Sex	
Female	154 (70.6)
Male	64 (29.4)
Race	
African American	6 (2.8)
Asian/Pacific Islander/Middle Eastern	6 (11.9)
Caucasian	169 (77.5)
Hispanic	5 (2.3)
Multiple races	8 (3.7)
Other	2 (0.9)
Prefer not to answer	2 (0.9)
Years in practice	
Still in training	35 (16.1)
<5 years	73 (33.5)
5–10 years	48 (22.0)
11–15 years	16 (7.3)
>15 years	46 (21.1)
Sexual orientation	
Straight/heterosexual	201 (92.2)
Lesbian/gay	12 (5.5)
Bisexual/pansexual/other	4 (1.8)
Prefer not to answer	1 (0.5)

### Family planning

The current number of children reported by men and women was statistically significantly different with a median of 2 (range: 0–5) for women and a median of 1.5 (range: 0–5) for men (*p* < 0.001, Table 2). No significant difference was seen for the number of children planned. However, a significantly higher proportion of women (51%) compared with men (19%) reported desire to have more children (*p* < 0.001) with no difference in reported ability to have more children (women 66% vs. men 75%; *p* > 0.99). A significantly higher proportion of women reported they would have had children earlier if they had a different job (75% vs. 45%, *p* < 0.001), and reported their career influenced the number of children they have or plan to have (56% vs. 24%, *p* < 0.001). While there was no significant difference in the proportion of women and men who delayed childbearing for professional or personal reasons, significantly more men delayed childbearing for financial reasons (39% vs. 18%, *p* = 0.02).

**Table 2.** Reproductive Planning and Experience by Gender

Variable	All (*N* = 218)	Females (*N* = 154)	Males (*N* = 64)	
	*n* (%)	*n* (%)	*n* (%)	*p*^a^
Reproductive planning				
Number of children planned Median (minimum, maximum)	2.0 (0.6)	2.0 (0.4)	2.0 (0.6)	0.11
Number of current children Median (minimum, maximum)	2.0 (0.5)	1.5 (0.5)	2.0 (0.5)	<0.001
Desire to have more children				
No	103 (48.4)	56 (37.1)	47 (75.8)	<0.001
Yes	89 (41.8)	77 (51.0)	12 (19.4)	
Do not know	21 (9.9)	18 (11.9)	3 (4.8)	
Ability to have more children				
No	12 (13.5)	11 (14.3)	1 (8.3)	>0.99
Yes	60 (67.4)	51 (66.2)	9 (75.0)	
Do not know	17 (19.1)	15 (19.5)	2 (16.7)	
Has career influenced the number of children you have/plan to have?				
No	92 (42.4)	51 (33.1)	41 (65.1)	<0.001
Yes	101 (46.5)	86 (55.8)	15 (23.8)	
I do not know	23 (10.6)	16 (10.4)	7 (11.1)	
Not applicable	1 (0.5)	1 (0.7)	0 (0.0)	
Would have had children sooner if you had a different job?				
No	70 (32.4)	37 (24.0)	33 (53.2)	<0.001
Yes	144 (66.7)	116 (75.3)	28 (45.2)	
Not applicable	2 (0.9)	1 (0.7)	1 (1.6)	
Why did you delay childbearing (check all that apply)				
Professional reasons	135 (93.8)	109 (94.0)	26 (92.9)	0.69
Financial reasons	32 (22.2)	21 (18.1)	11 (39.3)	0.02
Personal reasons	56 (38.9)	49 (42.2)	7 (25.0)	0.09
Other	2 (1.4)	2 (1.7)	0 (0.0)	>0.99
Reproductive experience				
Longest interval to pregnancy, conception or decision to stop trying?				
Currently trying to conceive	9 (5.4)	8 (6.8)	1 (2.0)	0.30
<1 year	90 (53.9)	63 (53.4)	27 (55.1)	
1–3 years	47 (28.1)	35 (29.7)	12 (24.5)	
>3 years	21 (12.6)	12 (10.2)	9 (18.4)	
Age at the time of first delivery				
<25 years	3 (2.0)	2 (1.9)	1 (2.3)	0.30
25–30 years	44 (29.7)	28 (26.9)	16 (36.4)	
31–34 years	54 (36.5)	38 (36.5)	16 (36.4)	
35–39 years	38 (25.7)	31 (29.8)	7 (15.9)	
40–44 years	9 (6.1)	5 (4.8)	4 (9.1)	
>45 years	0 (0.0)	0 (0.0)	0 (0.0)	
Stage of schooling at the time of first delivery				
Before medical school	2 (1.4)	2 (1.9)	0 (0.0)	0.01
Medical school/graduate school	7 (4.7)	2 (1.9)	5 (11.4)	
Post-doc	0 (0.0)	0 (0.0)	0 (0.0)	
Residency	42 (28.4)	24 (23.1)	18 (40.9)	
Fellowship	39 (26.4)	31 (29.8)	8 (18.2)	
After residency/fellowship	58 (39.2)	45 (43.3)	13 (29.5)	

^a^ *p*-Value is for Wilcoxon rank-sum test for continuous variables and Chi-square or Fisher's exact test for categorical variables, when appropriate.

### Reproductive experience

A majority of participants were able to conceive in less than 1 year (54%), with no difference by gender (Table 2). Thirty-two percent of respondents had their first child after 35 years of age, with no difference by gender. Nonetheless, men were more likely to have their first child during residency while women were more likely to delay until after completion of fellowship (*p* = 0.01).

### Infertility experience

Almost half of the female physicians (49%) reported having concerns about their fertility compared with 23% of males (*p* = 0.002, Table 3). Of those who had attempted to conceive and had concerns about their fertility, 81% (56/69) sought consultation for infertility, and all but 5 (91%, 51/56) subsequently underwent fertility treatment. A substantial proportion sought help during training, 25% during residency, and 21% during fellowship. Consistent with stage of training at the time of first child, men were more likely to seek infertility treatment during residency (75% vs. 17%; *p* = 0.002), and women were more likely to seek infertility treatment after completion of training (60% vs. 13%; *p* = 0.02).

**Table 3.** Infertility Experience

Variable	All	Females	Males	
	*n* (%)	*n* (%)	*n* (%)	*p*^a^
Fertility experience	(*N* = 166)	(*N* = 118)	(*N* = 48)	
Have you ever had concerns about your fertility?				
Yes	69 (41.6)	58 (49.2)	11 (22.9)	0.002
No	97 (58.4)	60 (50.8)	37 (77.1)	
Have you sought medical consultation for infertility?				
Yes	56 (81.2)	48 (82.8)	8 (72.7)	0.42
No	13 (18.8)	10 (17.2)	3 (27.3)	
Fertility treatment		(*N* = 48)	(*N* = 8)	
When did you seek fertility consultation? Check all that apply				
Before medical school	0 (0.0)	0 (0.0)	0 (0.0)	—
Medical school	1 (1.8)	0 (0.0)	1 (12.5)	0.14
Post-doc	1 (1.8)	1 (2.1)	0 (0.0)	>0.99
Residency	14 (25.0)	8 (16.7)	6 (75.0)	0.002
Fellowship	12 (21.4)	12 (25.0)	0 (0.0)	0.18
After fellowship	30 (53.6)	29 (60.4)	1 (12.5)	0.02
Was your program supportive of your fertility treatment (*e.g*., providing time and coverage for procedures)				
Not applicable	20 (35.7)	18 (37.5)	2 (25.0)	0.70
Very supportive	16 (28.6)	12 (25.0)	4 (50.0)	
Somewhat supportive	14 (25.0)	12 (25.0)	2 (25.0)	
Minimally supportive	4 (7.1)	4 (8.3)	0 (0.0)	
Not supportive	2 (3.6)	2 (4.2)	0 (0.0)	
Infertility support and psychological impact		(*N* = 58)	(*N* = 11)	
Did your colleagues know about your struggles with fertility or treatments?				
Yes—colleagues and program administration	6 (8.7)	5 (8.6)	1 (9.1)	0.93
Yes—only colleagues	29 (42.0)	25 (43.1)	4 (36.4)	
Yes—only program administration	2 (2.9)	2 (3.5)	0 (0.0)	
No	31 (44.9)	25 (43.1)	6 (54.5)	
Other	1 (1.4)	1 (1.7)	0 (0.0)	
Did you feel stigmatized for having an issue with infertility?				
Yes	9 (13.2)	7 (12.3)	2 (18.2)	0.63
No	59 (86.8)	50 (87.7)	9 (81.8)	
Have your infertility concerns resulted in depression?				
Yes	14 (20.3)	14 (24.1)	0 (0.0)	0.10
No	55 (79.7)	44 (75.9)	11 (100.0)	
Have your fertility concerns affected your work life?				
Yes	27 (39.7)	24 (42.1)	3 (27.3)	0.51
No	41 (60.3)	33 (57.9)	8 (72.7)	
Fertility preservation		(*N* = 154)	(*N* = 64)	
Have you considered oocyte/embryo cryopreservation?				
Yes	47 (24.5)	45 (31.9)	2 (3.9)	<0.001
No	145 (75.5)	96 (68.1)	49 (96.1)	
Have you sought consultation at a fertility center for fertility preservation?				
		(*N* = 44)	(*N* = 2)	
Yes	26 (56.5)	24 (54.5)	2 (100.0)	0.50
No	20 (43.5)	20 (45.5)	0 (0.0)	

^a^ *p*-Value is for Chi-square or Fisher's exact test for categorical variables, when appropriate.

Almost half (46%, 32/69) of the respondents who had fertility issues reported that they did not make their colleagues and/or administrators aware of their struggles with infertility. However, of those respondents who did inform their colleagues or administration, 81% (30/37) of the respondents reported that the program was very or somewhat supportive of their treatment in terms of providing time and coverage for fertility treatments. Thirteen percent felt stigmatized due to infertility struggles, 20% struggled with depression resulting from infertility concerns, and 40% of respondents reported fertility concerns affected their work life.

Over half (57%, 26/46) who had considered fertility preservation had sought consultation regarding oocyte and/or embryo cryopreservation, with no difference by gender (Table 3).

There were many physicians (27%) who responded to the open-ended question “Is there anything else you would like us to know about your fertility experience?” Multiple themes emerged, including (1) reasons for delayed childbearing; (2) stress due to infertility; (3) stigma of pregnancy; (4) optimal or suboptimal stage of training/career to start a family; and (5) barriers to infertility treatment or fertility preservation. Illustrative comments are provided in Table 4.

**Table 4.** Themes Which Emerged in Response to the Open-Ended Question “Is there anything else you would like us to know about your fertility experience?”

Reasons for delayed childbearing	“I delayed pregnancy because of residency. I could not imagine having a child during residency. I did a 2-year post-doc to improve my application for fellowship. I continued to delay, as I did not want to appear obviously pregnant while interviewing, or risk having morning sickness. Eventually when we attempted, we were unsuccessful for 1 year”
	“We are planning (embryo) transfer, but must wait until certain career events happen.”
	“I am postponing children to finish professional exams, which may impact my fertility in the future”
Stress due to infertility	“It was the most emotionally painful and psychologically difficult experience I have had in my life. Being an (ob)/gyn during our struggle made it significantly harder.”
	“Delayed attempted at conception until last few months of fellowship. We underwent 3 cycles of IVF and 3 surgeries in my first year of practice. This was a significant stressor in both my work and personal life.”
	“My fertility concerns did not result in depression but did affect my mood and concentration at work”
Stigma of pregnancy	“We did IVF and had to hide monitoring appointments and retrievals from partners.”
	“Interviewed for gyn onc fellowship while pregnant and I do think there is a cultural bias in our field against reproduction…imagine that [ART] would be really hard as even with ‘normal’ reproductive and pregnancy issues. I didn't talk about it, minimized, made sure as much as possible that it didn't impact how I functioned or was perceived as a trainee.
Optimal or suboptimal stage of training/career to start a family	“There was unspoken pressure not to have children during training… I believe residents and fellows should be encouraged and perhaps required to have intervening years of normal life to achieve personal goals throughout their training without giving up their long term goal of completing programs. Male and female roles in family life are different than they were in generations past. Perhaps medical education should catch up with the times.”
	“Due to my desire to avoid delivering during clinical years of fellowship training, I'm going to attempt pregnancy #2 now and my wife is having embryos frozen (which I will then carry) after fellowship.”
Barriers to infertility treatment or fertility preservation	“Wanted to cryopreserve embryos at age 28 but cost was prohibitive so got pregnant and delivered at age 30. Would have liked to delay childbearing further”
	“The cost of IVF was very hard to bear as a fellow in a State where it was not covered. IVF was covered in the state I did residency in.”
	“could not afford fertility preservation during training. now that i am done with training i am almost advanced maternal age. it does give me concern for future fertility.”

IVF, *in vitro* fertilization.

## Discussion

Our study showed that female gynecologic oncologists are significantly more likely to delay childbearing or change family planning due to their career choice than male gynecologic oncologists. There is also a high prevalence of infertility treatment and fertility preservation among gynecologic oncologists. These fertility struggles cause both professional and personal stress.

Delayed childbearing among female gynecologic oncologists is consistent with findings in other surgical specialties. The timing of pregnancy for surgeons has shifted toward late reproductive age, and usually after training. In a survey of 1950 female general or subspecialty surgeons, more than 50% of female surgeons delayed childbearing until they were in independent practice.^2^ The average age of first childbirth for 113 female thoracic surgeons was 34.3 years, whereas the national average was 25.4 years.^9,10^ In another survey, more than 80% of obstetrics and gynecology residents did not actively pursue pregnancy during residency.^11^ In comparison, more female physicians in nonprocedural fields were 30 years of age or younger at the time of first pregnancy compared with those in procedural or surgical fields (57% vs. 47%, *p* = 0.02).^12,13^ While these previously published studies focused on female surgeons/physicians, our study results support that there are differences in timing of childbearing by gender and not just specialty, as male gynecologic oncologists were more likely to start families during residency and females were more likely to delay until completion of fellowship. Interestingly males were more likely to report delaying due to financial concerns; our study was not designed to further explore whether this is due to a larger number of males starting families while receiving a limited residency salary, or whether this concern reinforces the gender stereotype of the male as the primary income generator.

Our study revealed that a career in gynecologic oncology influences the family planning decisions. Many studies have reported on the stigma associated with pregnancy for female physicians.^2,4,14^ In a survey of Plastic Surgery department chairs or residency program directors completed more than 20 years ago, 36% of respondents actively discouraged pregnancy during training due to concerns that pregnancy may impose great hardships on other residents and on the training program, as well as compromise her own training.^14^ Although the stigma of pregnancy during residency has decreased over time, it has not completely resolved. In a 2012 survey of 1950 female surgeons within 10 years of medical school graduation, 67% reported that stigma against pregnancy during training was still present.^2^ In a 2017 survey of 5782 female physicians, 35% reported maternal discrimination, and 90% of those physicians reported that discrimination was based on pregnancy or maternity leave.^15^

Our study showed a high prevalence of infertility among gynecologic oncologists. It took one or more years for 41% of the respondents to conceive or to decide to stop trying. In contrast, a survey of 327 female physicians from a variety of specialties reported a 24% infertility rate, with an average age at diagnosis of 34 years.^16^ In our study, among the 118 female gynecologic oncologists who have conceived or tried to conceive, 29% used *in vitro* fertilization, which is higher than the prevalence in the United States population (2%). This rate of assisted reproductive technology is similar to that reported in another survey of 400 women in procedural fields (23%).^12^

A survey conducted by the Society of Gynecologic Oncology showed that 32% of gynecologic oncologists experience burnout.^17^ This problem is of particular concern in this increasingly female-dominated field as female gender and younger age are both risk factors for burnout.^18^ Independently, infertility causes stress and can affect work life.^19,20^ A study evaluating the psychological impact of infertility showed that women with infertility had global psychological symptom scores (anxiety and depression) equivalent to individuals with cancer.^21^ Another survey of 3000 female physicians found that suffering from reproductive disorders (high-risk pregnancies and embryonic/fetal loss) was associated with depersonalization and personal accomplishment dimensions of burnout.^22^ Our study also showed that infertility caused stigmatization, depression, and affected work life. Almost 50% of the respondents who had struggles with fertility were not comfortable disclosing to their colleagues or administration. Given these findings and the prevalence of infertility among gynecologic oncologists, it is important for our professional society to address the stress of infertility and help create a culture and dialog to make it acceptable to discuss and disclose concerns.

This study on family planning, fertility, and infertility is unique in its focus on gynecologic oncology subspecialists. The strengths of our study are the national sample of practicing gynecologic oncologists from throughout the age spectrum and in different practice types. However, generalizability of the results is limited by the small sample size. Surgeons have historically low response rates to surveys, and previous studies have shown decreasing physician response rates of physicians to surveys overall, with results from a single-state study showing a similarly low 19% response rate.^23,24^ Results from other studies evaluating reasons for physician nonresponse showed the burden of receiving multiple survey requests and lack of time to be the primary reason for nonresponse (60%).^24^ Other reasons cited, which may be especially applicable to this study include lack of interest in the survey topic (13%) or perception the survey was requesting private information (8%). This study may be especially limited by selection bias, as those who have struggled with infertility or whose family planning has been altered by career choice may have been more motivated to complete the survey. This is further suggested by the majority of female response despite the fact that females comprise 46% of the Society of Gynecologic Oncology membership. Additionally, recall bias could influence the self-reported answers. While there are multiple studies on prevalence of infertility among physicians, there is a lack of data on prevalence of infertility treatment among physicians in other medical specialties, or within other career professions (*e.g*., lawyers), thus contextualization of our results is limited. We found significant differences by gender, which may represent social and/or biologic differences in family planning experiences, but validity of these results is limited by the disproportionately small number of men who completed the survey.

## Conclusions

Our study shows the difference in family planning and fertility among female and male gynecologic oncologists. Women have additional pressure since the optimal fertility period coincides with medical training and the early career period. With the increasing number of women in gynecologic oncology and surgical fields in general, it is critical that the medical profession recognize and address how family planning and infertility can contribute to physician burnout.

## Supplementary Material

Supplemental data

## Abbreviations Used

IVF: *in vitro* fertilization
REDCap: Research Electronic Data Capture
