# Supplementary material for: Differences in Family Planning and Fertility Among Female and Male Gynecologic Oncologists
Source: Womens Health Rep (New Rochelle). 2021 Apr 8;2(1):78–84. doi: 10.1089/whr.2020.0046 (PMC8080917; doi:10.1089/whr.2020.0046)
Supplement: Supplemental data [file Supp_Appendix.docx]

**Demographics**

What is your current age? 25 years or less


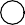

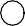

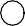

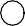

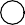


26-35 years

36-45 years

46-55 years

>55 years

What is your current gender identity? Female Male

Transgender Male/ Trans Man/ Female-to- Male (FTM) Transgender Female/ Trans Woman/ Male-to Female (MTF)


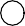

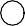

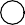

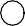


Gender queer, neither exclusively male of female Additional gender category/other


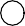

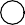

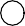


Prefer not to answer

Please specify

What is your race(s)? Check all that apply

Caucasian African-American Hispanic

Middle Eastern

Asian/ Pacific Islander Other

Prefer not to answer

Please specify

How many years have you been in practice? Still in training

< 5 years


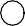

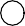

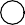

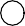

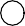


5-10 years

11-15 years

- 15 years

Do you think of yourself as: Straight/heterosexual Lesbian/Gay/homosexual Bisexual

Something else Don't know


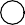

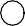

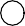

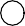

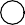

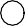


Prefer not to answer

What is your current partner status? Partnered Single

Divorced/Widowed Other


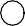

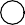

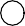

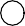

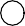


Prefer not to answer

Please specify

**Reproductive Planning**

**All questions apply to you and/or your partner(s).**

How many children (biological and non-biological) DID/DO you plan to have?

(Enter 999 if undecided)

How many children (biological and non-biological) DO you have?

Do you desire to have more children? Yes No

I don't know


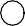

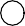

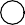


Do you think you CAN have more children? Yes No

I don't know


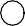

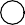

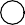


Do you think you would have had children sooner (or Yes

attempted) if you had a different job? No


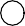

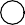

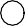


Not Applicable

Why did you delay childbearing?

Professional reasons Financial reasons Personal reasons Other

(Check all that apply)

Please specify

Has your career influenced the number of children you Yes have/plan to have? No

I don't know Not Applicable


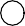

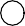

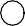

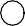


**Reproductive Experience**

**All questions apply to you and/or your partner(s).**

Have you or your current or previous partner(s) ever Yes


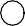

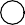


conceived or tried to conceive? No


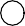

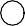


Do you have concerns about your future fertility? Yes No

What is the longest interval to pregnancy, conception Currently trying to conceive or decision to stop trying to conceive? < 1 year

1-3 years


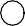

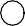

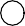

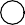


- 3 years

How long have you been trying to conceive? (months)

**What is your gravidity/parity?**

**(If your partner carried the pregnancies, how many pregnancies/pregnancy outcomes have you had)?**

Term

(Enter 0 if none)

Preterm

(Enter 0 if none)

Spontaneous abortion

(Enter 0 if none)

Induced abortion

(Enter 0 if none)

Reason for termination Unintended pregnancy/personal reasons Unintended pregnancy/professional commitments Unintended pregnancy/personal reasons+ professional commitments


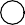
 Fetal anomaly


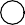

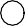

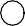


Gravida (calculated)

Live births

(Enter 0 if none)

Non-biological children

(Enter 0 if none)

Who carried your pregnancy/pregnancies? I did

My partner(s) did Both of us Neither of us


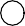

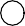

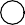

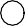


**For each child, indicate your age, geographic location, stage of schooling and practice at time**

**of birth.**

Child 1 - Age (years) at time of delivery

< 25

25-30

31-34

35-39

40-44

>45

Child 1 - Geographic Location at time of delivery
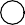
 East Coast (Connecticut, Maine, Massachusetts,

New Hampshire, Rhode Island, Vermont, Delaware, New Jersey, New York, Pennsylvania)


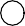
 East North Central (Illinois, Indiana, Michigan, Ohio, and Wisconsin)


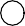
 West North Central (Iowa, Kansas, Minnesota, Missouri, Nebraska, North Dakota, South Dakota)


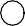
 South Atlantic (Florida, Georgia, Maryland, North Carolina, South Carolina, Virginia, District of Columbia, and West Virginia)


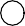
 South Central (Alabama, Kentucky, Mississippi, Tennessee, Arkansas, Louisiana, Oklahoma, and Texas)


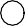
 West-Mountain (Arizona, Colorado, Idaho, Montana, Nevada, New Mexico, Utah, and Wyoming)


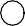
 West Pacific (Alaska, California, Hawaii, Oregon, and Washington)


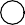
 Other

Child 1 - Stage of Schooling at time of delivery

Before medical school

Medical school/ Graduate school Post-doc

Residency Fellowship

After residency/fellowship

Child 1 - Practice Setting at time of delivery

Private practice

Private practice with teaching Hospital-based

Academic Other

(Check all that apply)

Child 2 - Age (years) at time of delivery

< 25

25-30

31-34

35-39

40-44

>45

Child 2 - Geographic Location at time of delivery
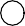
 East Coast (Connecticut, Maine, Massachusetts,

New Hampshire, Rhode Island, Vermont, Delaware, New Jersey, New York, Pennsylvania)


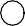
 East North Central (Illinois, Indiana, Michigan, Ohio, and Wisconsin)


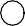
 West North Central (Iowa, Kansas, Minnesota, Missouri, Nebraska, North Dakota, South Dakota)


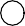
 South Atlantic (Florida, Georgia, Maryland, North Carolina, South Carolina, Virginia, District of Columbia, and West Virginia)


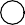
 South Central (Alabama, Kentucky, Mississippi, Tennessee, Arkansas, Louisiana, Oklahoma, and Texas)


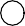
 West-Mountain (Arizona, Colorado, Idaho, Montana, Nevada, New Mexico, Utah, and Wyoming)


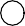
 West Pacific (Alaska, California, Hawaii, Oregon, and Washington)


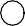
 Other

Child 2 - Stage of Schooling at time of delivery

Before medical school

Medical school/ Graduate school Post-doc

Residency Fellowship

After residency/fellowship

Child 2 - Practice Setting at time of delivery

Private practice

Private practice with teaching Hospital-based

Academic Other

(Check all that apply)

Child 3 - Age (years) at time of delivery

< 25

25-30

31-34

35-39

40-44

>45

Child 3 - Geographic Location at time of delivery
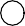
 East Coast (Connecticut, Maine, Massachusetts,

New Hampshire, Rhode Island, Vermont, Delaware, New Jersey, New York, Pennsylvania)


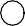
 East North Central (Illinois, Indiana, Michigan, Ohio, and Wisconsin)


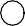
 West North Central (Iowa, Kansas, Minnesota, Missouri, Nebraska, North Dakota, South Dakota)


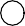
 South Atlantic (Florida, Georgia, Maryland, North Carolina, South Carolina, Virginia, District of Columbia, and West Virginia)


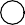
 South Central (Alabama, Kentucky, Mississippi, Tennessee, Arkansas, Louisiana, Oklahoma, and Texas)


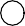
 West-Mountain (Arizona, Colorado, Idaho, Montana, Nevada, New Mexico, Utah, and Wyoming)


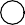
 West Pacific (Alaska, California, Hawaii, Oregon, and Washington)


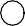
 Other

Child 3 - Stage of Schooling at time of delivery

Before medical school

Medical school/ Graduate school Post-doc

Residency Fellowship

After residency/fellowship

Child 3 - Practice Setting at time of delivery

Private practice

Private practice with teaching Hospital-based

Academic Other

(Check all that apply)

Child 4 - Age (years) at time of delivery

< 25

25-30

31-34

35-39

40-44

>45

Child 4 - Geographic Location at time of delivery
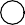
 East Coast (Connecticut, Maine, Massachusetts,

New Hampshire, Rhode Island, Vermont, Delaware, New Jersey, New York, Pennsylvania)


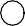
 East North Central (Illinois, Indiana, Michigan, Ohio, and Wisconsin)


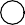
 West North Central (Iowa, Kansas, Minnesota, Missouri, Nebraska, North Dakota, South Dakota)


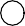
 South Atlantic (Florida, Georgia, Maryland, North Carolina, South Carolina, Virginia, District of Columbia, and West Virginia)


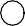
 South Central (Alabama, Kentucky, Mississippi, Tennessee, Arkansas, Louisiana, Oklahoma, and Texas)


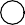
 West-Mountain (Arizona, Colorado, Idaho, Montana, Nevada, New Mexico, Utah, and Wyoming)


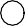
 West Pacific (Alaska, California, Hawaii, Oregon, and Washington)


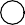
 Other

Child 4 - Stage of Schooling at time of delivery

Before medical school

Medical school/ Graduate school Post-doc

Residency Fellowship

After residency/fellowship

Child 4 - Practice Setting at time of delivery

Private practice

Private practice with teaching Hospital-based

Academic Other

(Check all that apply)

Child 5 - Age (years) at time of delivery

< 25

25-30

31-34

35-39

40-44

>45

Child 5 - Geographic Location at time of delivery
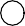
 East Coast (Connecticut, Maine, Massachusetts,

New Hampshire, Rhode Island, Vermont, Delaware, New Jersey, New York, Pennsylvania)


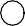
 East North Central (Illinois, Indiana, Michigan, Ohio, and Wisconsin)


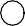
 West North Central (Iowa, Kansas, Minnesota, Missouri, Nebraska, North Dakota, South Dakota)


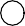
 South Atlantic (Florida, Georgia, Maryland, North Carolina, South Carolina, Virginia, District of Columbia, and West Virginia)


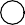
 South Central (Alabama, Kentucky, Mississippi, Tennessee, Arkansas, Louisiana, Oklahoma, and Texas)


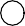
 West-Mountain (Arizona, Colorado, Idaho, Montana, Nevada, New Mexico, Utah, and Wyoming)


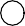
 West Pacific (Alaska, California, Hawaii, Oregon, and Washington)


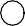
 Other

Child 5 - Stage of Schooling at time of delivery

Before medical school

Medical school/ Graduate school Post-doc

Residency Fellowship

After residency/fellowship

Child 5 - Practice Setting at time of delivery

Private practice

Private practice with teaching Hospital-based

Academic Other

(Check all that apply)

Child 6 - Age (years) at time of delivery

< 25

25-30

31-34

35-39

40-44

>45

Child 6 - Geographic Location at time of delivery
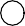
 East Coast (Connecticut, Maine, Massachusetts,

New Hampshire, Rhode Island, Vermont, Delaware, New Jersey, New York, Pennsylvania)


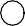
 East North Central (Illinois, Indiana, Michigan, Ohio, and Wisconsin)


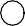
 West North Central (Iowa, Kansas, Minnesota, Missouri, Nebraska, North Dakota, South Dakota)

South Atlantic (Florida, Georgia, Maryland, North Carolina, South Carolina, Virginia, District of Columbia, and West Virginia)

South Central (Alabama, Kentucky, Mississippi, Tennessee, Arkansas, Louisiana, Oklahoma, and Texas)

West-Mountain (Arizona, Colorado, Idaho, Montana, Nevada, New Mexico, Utah, and Wyoming)

West Pacific (Alaska, California, Hawaii, Oregon, and Washington)

Other

Child 6 - Stage of Schooling at time of delivery

Before medical school

Medical school/ Graduate school Post-doc

Residency Fellowship

After residency/fellowship

Child 6 - Practice Setting at time of delivery

Private practice

Private practice with teaching Hospital-based

Academic Other

(Check all that apply)

Child 7 - Age (years) at time of delivery

< 25

25-30

31-34

35-39

40-44

>45

Child 7 - Geographic Location at time of delivery East Coast (Connecticut, Maine, Massachusetts,

New Hampshire, Rhode Island, Vermont, Delaware, New Jersey, New York, Pennsylvania)

East North Central (Illinois, Indiana, Michigan, Ohio, and Wisconsin)

West North Central (Iowa, Kansas, Minnesota, Missouri, Nebraska, North Dakota, South Dakota)

South Atlantic (Florida, Georgia, Maryland, North Carolina, South Carolina, Virginia, District of Columbia, and West Virginia)

South Central (Alabama, Kentucky, Mississippi, Tennessee, Arkansas, Louisiana, Oklahoma, and Texas)

West-Mountain (Arizona, Colorado, Idaho, Montana, Nevada, New Mexico, Utah, and Wyoming)

West Pacific (Alaska, California, Hawaii, Oregon, and Washington)

Other

Child 7 - Stage of Schooling at time of delivery

Before medical school

Medical school/ Graduate school Post-doc

Residency Fellowship

After residency/fellowship

Child 7 - Practice Setting at time of delivery

Private practice

Private practice with teaching Hospital-based

Academic Other

(Check all that apply)

Child 8 - Age (years) at time of delivery

< 25

25-30

31-34

35-39

40-44

>45

Child 8 - Geographic Location at time of delivery East Coast (Connecticut, Maine, Massachusetts,

New Hampshire, Rhode Island, Vermont, Delaware, New Jersey, New York, Pennsylvania)

East North Central (Illinois, Indiana, Michigan, Ohio, and Wisconsin)

West North Central (Iowa, Kansas, Minnesota, Missouri, Nebraska, North Dakota, South Dakota)

South Atlantic (Florida, Georgia, Maryland, North Carolina, South Carolina, Virginia, District of Columbia, and West Virginia)

South Central (Alabama, Kentucky, Mississippi, Tennessee, Arkansas, Louisiana, Oklahoma, and Texas)

West-Mountain (Arizona, Colorado, Idaho, Montana, Nevada, New Mexico, Utah, and Wyoming)

West Pacific (Alaska, California, Hawaii, Oregon, and Washington)

Other

Child 8 - Stage of Schooling at time of delivery

Before medical school

Medical school/ Graduate school Post-doc

Residency Fellowship

After residency/fellowship

Child 8 - Practice Setting at time of delivery

Private practice

Private practice with teaching Hospital-based

Academic Other

(Check all that apply)

Child 9 - Age (years) at time of delivery

< 25

25-30

31-34

35-39

40-44

>45

Child 9 - Geographic Location at time of delivery East Coast (Connecticut, Maine, Massachusetts,

New Hampshire, Rhode Island, Vermont, Delaware, New Jersey, New York, Pennsylvania)

East North Central (Illinois, Indiana, Michigan, Ohio, and Wisconsin)

West North Central (Iowa, Kansas, Minnesota, Missouri, Nebraska, North Dakota, South Dakota)

South Atlantic (Florida, Georgia, Maryland, North Carolina, South Carolina, Virginia, District of Columbia, and West Virginia)

South Central (Alabama, Kentucky, Mississippi, Tennessee, Arkansas, Louisiana, Oklahoma, and Texas)

West-Mountain (Arizona, Colorado, Idaho, Montana, Nevada, New Mexico, Utah, and Wyoming)

West Pacific (Alaska, California, Hawaii, Oregon, and Washington)

Other

Child 9 - Stage of Schooling at time of delivery

Before medical school

Medical school/ Graduate school Post-doc

Residency Fellowship

After residency/fellowship

Child 9 - Practice Setting at time of delivery

Private practice

Private practice with teaching Hospital-based

Academic Other

(Check all that apply)

Child 10 - Age (years) at time of delivery

< 25

25-30

31-34

35-39

40-44

>45

Child 10 - Geographic Location at time of delivery East Coast (Connecticut, Maine, Massachusetts,

New Hampshire, Rhode Island, Vermont, Delaware, New Jersey, New York, Pennsylvania)

East North Central (Illinois, Indiana, Michigan, Ohio, and Wisconsin)

West North Central (Iowa, Kansas, Minnesota, Missouri, Nebraska, North Dakota, South Dakota)

South Atlantic (Florida, Georgia, Maryland, North Carolina, South Carolina, Virginia, District of Columbia, and West Virginia)

South Central (Alabama, Kentucky, Mississippi, Tennessee, Arkansas, Louisiana, Oklahoma, and Texas)

West-Mountain (Arizona, Colorado, Idaho, Montana, Nevada, New Mexico, Utah, and Wyoming)

West Pacific (Alaska, California, Hawaii, Oregon, and Washington)

Other

Child 10 - Stage of Schooling at time of delivery

Before medical school

Medical school/ Graduate school Post-doc

Residency Fellowship

After residency/fellowship

Child 10 - Practice Setting at time of delivery

Private practice

Private practice with teaching Hospital-based

Academic Other

(Check all that apply)

Child 11 - Age (years) at time of delivery

< 25

25-30

31-34

35-39

40-44

>45

Child 11 - Geographic Location at time of delivery East Coast (Connecticut, Maine, Massachusetts,

New Hampshire, Rhode Island, Vermont, Delaware, New Jersey, New York, Pennsylvania)

East North Central (Illinois, Indiana, Michigan, Ohio, and Wisconsin)

West North Central (Iowa, Kansas, Minnesota, Missouri, Nebraska, North Dakota, South Dakota)

South Atlantic (Florida, Georgia, Maryland, North Carolina, South Carolina, Virginia, District of Columbia, and West Virginia)

South Central (Alabama, Kentucky, Mississippi, Tennessee, Arkansas, Louisiana, Oklahoma, and Texas)

West-Mountain (Arizona, Colorado, Idaho, Montana, Nevada, New Mexico, Utah, and Wyoming)

West Pacific (Alaska, California, Hawaii, Oregon, and Washington)

Other

Child 11 - Stage of Schooling at time of delivery

Before medical school

Medical school/ Graduate school Post-doc

Residency Fellowship

After residency/fellowship

Child 11 - Practice Setting at time of delivery

Private practice

Private practice with teaching Hospital-based

Academic Other

(Check all that apply)

**Fertility experience**

Have you ever had concerns about your fertility? Yes No

Have you sought infertility help from a fertility Yes

specialist? No

When did you seek fertility consultation?

Before medical school Medical School

Post-doc Residency Fellowship

After Fellowship (Check all that apply)

What fertility treatment(s) have you attempted?

None

Oocyte Cryopreservation Intrauterine insemination (IUI) In vitro fertilization (IVF)

IVF with Preimplantation genetic testing Other

(Check all that apply)

Please specify

What were the reasons you did not seek infertility I was/am not interested

treatment? Interested but do not have time

Interested but partner is not interested Still considering my options

Other

Please specify

Did your colleagues know about your struggles with Yes (colleagues and program administration) fertility or treatments? Yes (only colleagues)

Yes (only program administration) No

Other

Please specify

Did you feel stigmatized for having an issue with Yes

fertility? No

Other

Please specify

Have your infertility concerns resulted in Yes

depression? No

Have your fertility concerns affected your work life? Yes No

Was your program supportive of you going through the Not applicable treatment? (providing time and coverage for Very supportive

important procedures) Somewhat supportive

Minimally supportive Not supportive

Have you considered oocyte/embryo cryopreservation Yes for fertility preservation? No

Have you had a consultation at a fertility center Yes

regarding oocyte/embryo cryopreservation? No Other

Please specify

Is there anything else you would like us to know about your fertility experience?
